# Trends in incidence, prevalence, and mortality of neuromuscular disease in Ontario, Canada: A population-based retrospective cohort study (2003-2014)

**DOI:** 10.1371/journal.pone.0210574

**Published:** 2019-03-26

**Authors:** Louise Rose, Douglas McKim, David Leasa, Mika Nonoyama, Anu Tandon, Yu Qing Bai, Reshma Amin, Sherri Katz, Roger Goldstein, Andrea Gershon

**Affiliations:** 1 Department of Critical Care, Sunnybrook Health Sciences Centre and Sunnybrook Research Institute, Toronto, Canada; 2 Florence Nightingale Faculty of Nursing, Midwifery and Palliative Care, King’s College London, London, United Kingdom; 3 Lawrence S. Bloomberg Faculty of Nursing, University of Toronto, Toronto, Canada; 4 Institute of Clinical Evaluative Sciences, Toronto, Canada; 5 The Ottawa Hospital Respiratory Rehabilitation and The Ottawa Hospital Sleep Centre and Ottawa Hospital Research Institute, Ottawa, Canada; 6 Faculty of Medicine, University of Ottawa, Ottawa, Canada; 7 Department of Medicine, Divisions of Critical Care and Respirology, London Health Sciences Centre, London, Canada; 8 Faculty of Medicine, Western University, London, Canada; 9 Faculty of Health Sciences, University of Ontario Institute of Technology, Oshawa, Canada; 10 Hospital for Sick Children (SickKids) Research Institute, Toronto, Canada; 11 Rehabilitation Sciences Institute, University of Toronto, Toronto, Canada; 12 Department of Respirology & Clinical Immunology, Sunnybrook Health Sciences Centre and Sunnybrook Research Institute, Toronto, Canada; 13 Faculty of Medicine, University of Toronto, Toronto, Canada; 14 Institute of Health Policy, Management and Evaluation, University of Toronto, Toronto, Canada; 15 Children’s Hospital of Eastern Ontario and Children’s Hospital of Eastern Ontario Research Institute, Ottawa, Canada; 16 West Park Healthcare Centre, Toronto, Canada; Ottawa Hospital Research Institute, CANADA

## Abstract

**Background:**

Population trends of disease prevalence and incidence over time measure burden of disease and inform healthcare planning. Neuromuscular disorders (NMD) affect muscle and nerve function with varying degrees of severity and disease progression.

**Objective:**

Using health administrative databases we described trends in incidence, prevalence, and mortality of adults and children with NMD. We also explored place of death and use of palliative care.

**Methods:**

Population-based (Ontario, Canada) cohort study (2003 to 2014) of adults and children with NMD identified using International Classification of Disease and health insurance billing codes within administrative health databases.

**Results:**

Adult disease prevalence increased on average per year by 8% (95% confidence interval (CI) 6% to 10%, P <.001), with the largest increase in adults18-39 years. Childhood disease prevalence increased by 10% (95% CI 8% to 11%, P <.0001) per year, with the largest increase in children 0 to 5 years. Prevalence increased across all diagnoses except amyotrophic lateral sclerosis and spinal muscular atrophy for adults and all diagnoses for children. Adult incidence decreased by 3% (95% CI -4% to -2%, P <.0001) but incidence remained stable in children. Death occurred in 34,336 (18.5%) adults; 21,236 (61.8%) of whom received palliative care. Death occurred in 1,009 (5.6%) children; 507 (50.2%) of whom received palliative care. Mortality decreased over time in adults (odds ratio (OR) 0.86, 95% CI 0.86–0.87, P <.0001) and children (OR 0.79, 95% CI 0.76–0.82, P <.0001). Use of palliative care over time increased for adults (OR 1.18, 95% CI 1.09 to 1.28, P <.0001) and children (OR 1.22, 95% CI 1.20 to 1.23, P <.0001).

**Conclusions:**

In both adults and children, NMD prevalence is rising and mortality rates are declining. In adults incidence is decreasing while in children it remains stable. This confirms on a population-based level the increased survival of children and adults with NMD.

## Introduction

Neuromuscular disorders (NMD) vary in severity and disease progression [1, 2]. These disorders result in longstanding functional deficits that result in substantial utilization of healthcare resources in addition to the emotional, financial and social burden to those affected individuals and their families. Some forms of NMD are hereditary and manifest at birth or early childhood whereas the incidence of other forms increases with age [3]. On an international level, an aging population means the prevalence of NMD is likely to increase.

Although some estimates of NMD prevalence exist [4, 5], case ascertainments methods are highly variable and frequently biased. These include chart reviews, surveys, family histories, interviews with relatives and patient registries [4]. Few studies are conducted using population based health administration databases containing all publicly funded healthcare encounters for a region or an entire country. Population based estimates of disease prevalence and trends over time inform an understanding of the natural history as well as the impact of newer management interventions on the epidemiology of disease measure burden of disease and inform healthcare planning [6].

Understanding the magnitude of NMD at a population level is important to understand the impact on the healthcare system and the healthcare burden of individuals to inform healthcare policy. Therefore, we conducted a population based cohort study using health administrative databases to determine the prevalence, incidence, and mortality for adults and children with NMD. Our secondary objectives were to describe prevalence and incidence for various types of NMD, location of death, and use of palliative care services.

## Methods

### Study design

We conducted a retrospective population-based study using health administrative databases for the province of Ontario, Canada held at the Institute for Clinical Evaluative Sciences (ICES) using unique encoded identifiers to link databases. These databases contain anonymized data for all residents of the province of Ontario, population approximately 13 million [7]. In Canada, universal public health insurance funded through general taxation covers cost of all medically necessary care.

### Population and data sources

**Fig 1:**
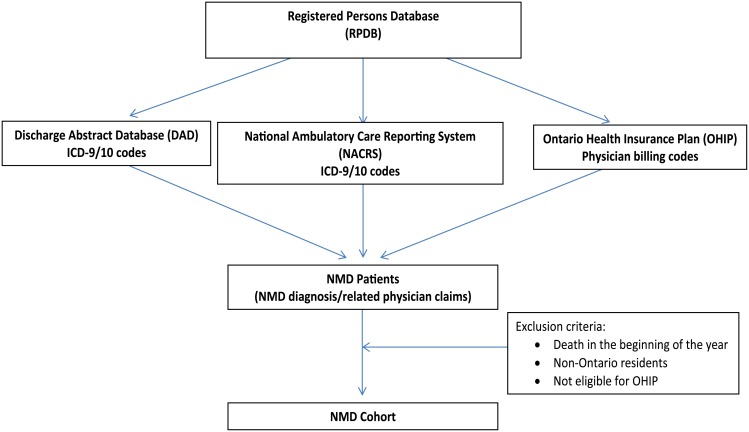
Schematic of cohort creation.

Form the Ontario Registered Persons Database (RPDB)we identified adults aged 18 to 105 and children aged 0 to <18 years with NMD using hospital diagnostic codes (International Classification of Disease (ICD)-9, ICD-10) or physician billing (Ontario Health Insurance Plan (OHIP)) codes *a priori* considered specific to NMD within health administration databases. We verified physician billing codes for NMD with neurologists working with research team members. We sought the first instance (from fiscal year 2003 onwards) of these codes considered the ‘most responsible’ or secondary contributing diagnoses indicating hospital admission with a NMD diagnosis in the Discharge Abstract Database. This database includes all hospitalizations including demographic, procedural data (up to 24 comorbidities and procedures), and in-hospital death [8]. We also examined hospital diagnostic codes in the National Ambulatory Care Reporting System containing all emergency department visits and physician billings codes in the OHIP database [9] that contains all physician billings including procedures (Fig 1). To establish death outside of hospital we linked to the RPDB. To determine use of palliative care we sought validated palliative care codes [10] in the the hospitalization, ambulatory care and physician databases as well as the Continuing Care Reporting System, the Complex Continuing Care database, and the Home Care Database.

For individuals with the first occurrence of a NMD diagnostic code found using a physician billing code, we sought to confirm NMD diagnosis by looking backwards and forwards in time (1998–2014) in the hospitalization database for a NMD-related hospital admission identified through an ICD code. For individuals identified using the physician billing code 349 which is used as a non-specific catch all code for NMD, we again looked backwards and forwards in the hospitalization (1998–2014) and ambulatory care (2000–2014) databases and sought ICD codes and physician billings from a neurologist or for an electromyogram to increase our certainty the patient had NMD.

We grouped hospital diagnostic and physician NMD billing codes into 12 categories: amyotrophic lateral sclerosis/motor neurone disease; cerebral palsy [11]; Guillain-Barré syndrome [12]; metabolic disorders; multiple sclerosis [13]; muscular dystrophy; myasthenia gravis [14]; neuromuscular disorders (non-specific diagnoses); neuropathy; post-polio syndrome; spina bifida; and spinal muscular atrophy. Wherever possible, we used codes previously reported in studies specific to these disease categories (see S1 File for disease specific codes).

### Outcomes

We established annual prevalence and annual incidence considering all adults and all children with a NMD diagnosis, and separately for those adults and children identified through hospitalization or ED presentation during that year. We also established annual prevalence and incidence for each NMD disease category. However, due to inability to classify disease type further with identification of NMD using physician billing we restricted our estimates of prevalence and incidence to those individuals with case ascertainment through hospital and ED diagnostic (ICD) codes. To establish incidence, we used a five-year look back window to confirm the individual had not received any health services for NMD previously and calculated annual incidence from 1^st^ April 2008 to 31^st^ March 2015. Individuals with prior health services utilization for NMD in these years were counted as prevalent but not incident. We established all-cause mortality rates, identified death location (in or outside a hospital), and determined use of palliative care.

### Ethical considerations

We conducted our study according to a pre-specified protocol approved by the Research Ethics Board at Sunnybrook Health Sciences Centre, Toronto, Ontario and according to privacy regulations of the Institute for Clinical Evaluative Sciences.

### Statistical analyses

We estimated annual prevalence by dividing the number of affected individuals alive at the end of each fiscal year by the census population estimated in the corresponding year from the RPDB. To estimate annual incidence we divided the number of new cases by the number of individuals at risk for NMD, considered as all adults resident in the province of Ontario without NMD, also from the RPDB estimated census population. Using patient level data we examined linear trends of prevalence and incidence (overall and by age and sex categories) over time using linear regression models with year as an independent variable [15]. We examined trends in mortality and proportion of adults and children receiving palliative care using logistic regression. We conducted analyses in SAS Enterprise Guide 7.1 (SAS Institute Inc., Cary, NC, USA).

## Results

### Prevalence

**Fig 2:**
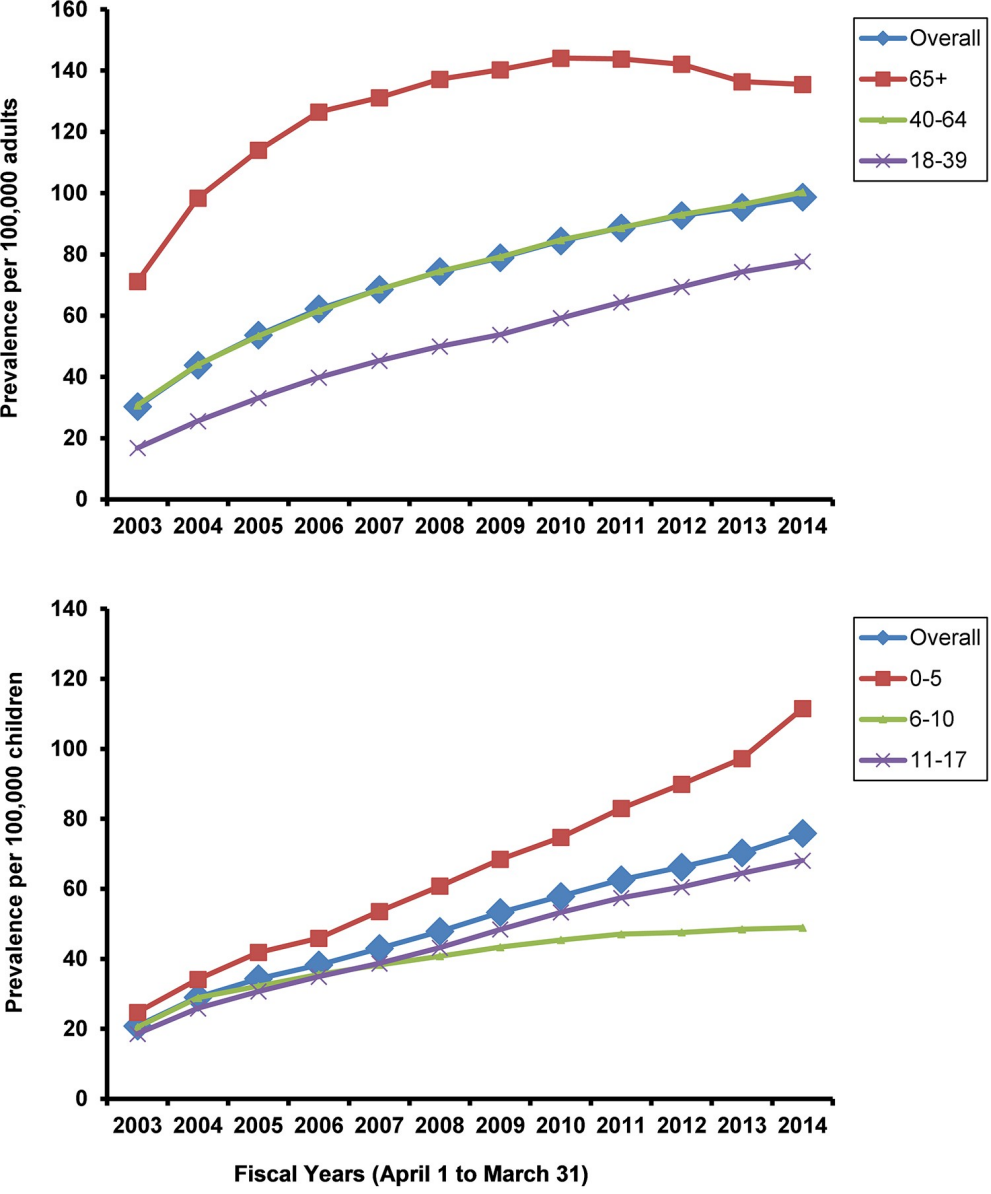
Prevalence of neuromuscular disease per 100,000 adults and per 100,000 children for individuals whose index case was identified through ED presentation or hospital admission (i.e., excludes those identified through billing codes).

For adults, the crude prevalence of NMD considering only those identified through an ED presentation or hospital admission averaged over the 12-year period (Fiscal years 2003–2014) was 72.6/100,000 adults whereas it was 1,344/100,000 adults when including those identified through physician billing (See Table A in S1 File for total eligible patient population and NMD prevalent cases). Prevalence of patients identified through ED presentation or hospital admission increased on average per year by 8% (95% CI 6% to 10%, P <.0001 for linear trend). Prevalence increased across all diagnoses except ALS and SMA (Table 1). Although prevalence was highest in adults aged ≥ 65 years and increased for all adult age categories, the greatest average increase was in younger adults: 18–39 years 11% per year (95% CI 10% to 11%) P <.0001; 40–64 8% per year (95% CI 7% to 9%) P <.0001 and in adults aged ≥65 years 4% per year (95% CI 2% to 5%, P <.0001) (Fig 2). Prevalence increased by 8% (95% CI 7% to 8%) in both males and females (Table B in S1 File).

**Table 1:** Prevalence by year^a^.

Overall (yrs)		2003	2004	2005	2006	2007	2008	2009	2010	2011	2012	2013	2014	Rate^b^	95% CI	P^c^
Adults																
All NMD diagnoses	72.7	30.3	43.9	53.7	62.2	68.6	74.4	78.9	84.4	88.7	92.7	95.4	98.7	0.08	0.07,0.09	<.0001
Multiple sclerosis	18.8	9.4	12.7	14.7	16.4	17.9	19.2	20.0	21.1	21.9	23.0	24.0	24.9	0.07	0.06,0.08	<.0001
Cerebral palsy	9.8	3.9	6.0	7.4	8.7	9.6	10.4	10.9	11.5	11.8	12.1	12.3	12.6	0.07	0.06,0.09	<.0001
Neuropathies	8.2	2.9	4.4	5.5	6.5	7.3	8.0	8.9	9.7	10.2	11.0	11.5	12.2	0.10	0.09,0.11	<.0001
Guillian Barré	7.5	1.4	2.9	3.9	5.1	6.2	7.2	8.2	9.1	10.3	11.1	11.8	12.6	0.14	0.13,0.14	<.0001
Muscular dystrophy	6.5	2.8	4.0	5.0	5.9	6.5	6.8	7.0	7.6	7.9	8.2	8.3	8.6	0.07	0.06,0.09	<.0001
Metabolic disease	6.0	2.2	3.9	5.0	5.7	6.1	6.8	6.9	6.9	7.1	7.1	6.9	6.8	0.06	0.04,0.08	<.0001
NMD (non-specific)	5.8	2.3	3.2	4.3	5.1	5.4	5.7	6.1	6.7	7.0	7.5	7.7	7.9	0.08	0.07,0.09	<.0001
Spina bifida	4.6	1.9	2.7	3.3	3.9	4.5	4.9	5.1	5.5	5.8	5.9	6.1	6.0	0.08	0.07,0.09	<.0001
Myasthenia gravis	2.7	1.4	1.8	2.1	2.6	2.7	2.8	2.9	3.1	3.2	3.3	3.5	3.5	0.07	0.05,0.08	<.0001
ALS/MND	2.3	2.2	2.3	2.2	2.3	2.4	2.3	2.4	2.4	2.6	2.4	2.1	2.1	0.00	-0.01,0.01	0.9759
Post-polio syndrome	0.4	0.02	0.02	0.03	0.03	0.03	0.05	0.3	0.5	0.8	0.9	1.1	1.3	0.29	0.23,0.36	<.0001
SMA	<0.2	<0.2	<0.2	<0.2	<0.2	0.2	0.2	0.2	0.2	0.2	0.2	0.2	<0.2	0.05	0.00,0.09	0.0378
Children																
All NMD diagnoses	50.0	20.9	29.1	34.3	38.3	43.0	47.9	53.3	57.9	62.7	66.3	70.3	75.9	0.10	0.09,0.10	<.0001
Cerebral palsy	19.5	11.2	14.5	16.3	17.7	18.8	20.1	20.8	21.6	22.2	22.7	23.5	24.1	0.05	0.05,0.06	<.0001
Guillian Barré	3.4	0.6	1.0	1.4	1.7	2.2	2.9	3.7	4.3	4.8	5.4	5.8	6.4	0.16	0.16,0.17	<.0001
Muscular dystrophy	3.8	1.9	2.4	2.8	3.1	3.2	3.7	4.3	4.4	4.8	4.9	5.0	5.6	0.08	0.08,0.09	<.0001
Metabolic disease	6.3	2.2	3.5	4.1	4.5	5.5	6.0	6.6	7.3	7.8	8.6	9.1	9.9	0.11	0.10,0.11	<.0001
Myasthenia gravis	0.5	<0.2	<0.2	0.2	0.3	0.4	0.5	0.5	0.7	0.8	0.8	1.0	1.1	0.17	0.16,0.19	<.0001
ALS/MND	<0.2	<0.2	<0.2	<0.2	<0.2	<0.2	<0.2	<0.2	<0.2	<0.2	<0.2	<0.2	<0.2	0.12	0.08,0.15	<.0001
Multiple sclerosis	4.3	0.7	1.4	1.9	2.3	3.0	3.6	4.6	5.1	5.9	6.6	7.4	8.5	0.16	0.15,0.17	<.0001
NMD (non-specific)	1.9	0.5	0.8	0.9	1.3	1.5	1.8	2.0	2.3	2.7	2.8	3.1	3.5	0.14	0.13,0.14	<.0001
Neuropathies	1.4	0.4	0.8	1.0	1.2	1.3	1.3	1.4	1.6	1.7	1.8	1.9	2.1	0.10	0.08,0.11	<.0001
Spina bifida	8.2	2.9	4.0	4.9	5.6	6.5	7.4	8.5	9.8	11.0	11.7	12.6	13.6	0.12	0.12,0.12	<.0001
SMA	0.7	0.4	0.6	0.7	0.6	0.6	0.6	0.7	0.7	0.8	0.9	0.7	0.8	0.04	0.02,0.06	<.0001

^a^ Prevalence/100,000 individuals whose index case was identified through ED presentation or hospital admission (i.e., excludes those identified through physician billing only).

^b^ Average change in prevalence per year.

^c^ P value for linear trend.

ALS: amyotrophic lateral sclerosis; MND: motor neurone disease; NMD: neuromuscular disease (non-specific diagnosis); SMA: spinal muscular atrophy.

For children, crude prevalence of NMD over the 12-year period (fiscal years 2003–2014) was 50.0/100,000 children considering only those identified through ED presentation or hospital admission whereas it was 544/100,000 children when also including those identified through physician billing. Prevalence increased on average per year by 10% (95% CI 8% to 11%, P <.001 linear trend). Prevalence increased across all diagnoses (Table 1).Prevalence increased for all child age categories, though greatest average increase was in the youngest category: 0 to 5 years 11% per year (95% CI 11% to 12%) P <.0001; 6 to10 6% per year (95% CI 5% to 7%) P <.0001 and aged 11 to 17 10% per year (95% CI 9% to 10%) P <.0001 (Fig 2). Prevalence increased slightly more in girls, 10% (95% CI 9% to 10%) than boys, 9% (95% CI 9% to 10%) (Table B in S1 File).

### Incidence

**Fig 3:**
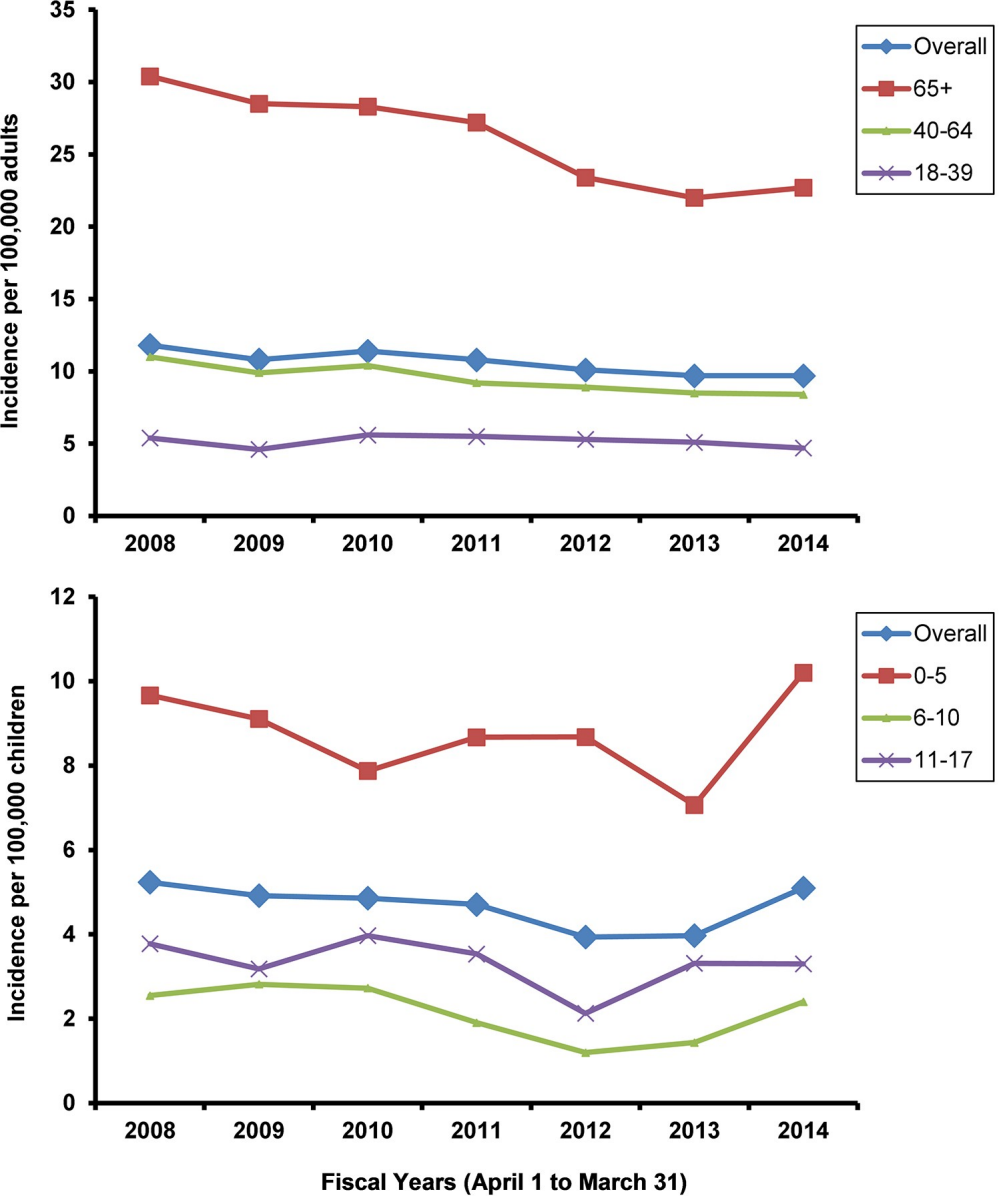
Incidence of neuromuscular disease per 100,000 adults and per 100,000 children for individuals whose index case was identified through ED presentation or hospital admission (i.e., excludes those identified through billing codes).

For adults, the overall incidence of NMD over the 7-year period (2008–2014) was 10.6/100,000 adults considering only those identified through ED presentation or hospital admission and 182/100,000 adults when including those identified through physician billing. Incidence decreased on average per year by 3% (95% CI -4% to -2%, P <.0001 for linear trend). Incidence decreased over time for multiple sclerosis, Guillian Barré syndrome, cerebral palsy, post-polio syndrome and SMA (Table 2). Incidence did not change in adults aged 18–39 (-1%, 95% CI -3%, 2%, P = 0.45) but decreased in adults aged 40–64 (-5%, 95% CI -6% to -3%, P<0.001) and in adults aged 65 and over (-6%, 95% CI -7% to -4%, P<0.001) (Fig 3). Incidence decreased by 3% (95% CI -4% to -2%) in females and by 4% (95% CI -4% to -5%) in males (Table C in S1 File).

**Table 2:** Incidence by year^a^.

Overall (yrs)		2008	2009	2010	2011	2012	2013	2014	Rate^b^	95% CI	P^c^
Adults											
All NMD diagnoses	10.6	11.8	10.8	11.4	10.8	10.1	9.7	9.7	-0.03	-0.04,-0.02	<.0001
Multiple sclerosis	2.0	2.3	1.8	2.0	1.8	2.1	2.0	2.0	-0.01	-0.04,0.02	0.6319
Cerebral palsy	1.0	1.3	1.1	1.1	0.9	0.8	0.8	0.8	-0.09	-0.11,-0.06	<.0001
Neuropathies	1.5	1.4	1.5	1.6	1.3	1.5	1.4	1.5	0.00	-0.02,0.02	0.9999
Guillian Barré	1.3	1.3	1.3	1.4	1.5	1.2	1.2	1.4	0.003	-0.03,0.03	0.8541
Muscular dystrophy	1.1	1.1	1.0	1.2	1.1	1.0	1.0	1.0	-0.02	-0.04,0.01	0.1367
Metabolic disease	0.9	1.6	0.8	0.9	0.8	0.7	0.7	0.7	-0.13	-0.21,-0.04	0.0037
NMD (non-specific)	1.2	1.1	1.3	1.3	1.2	1.2	1.2	1.0	-0.02	-0.05,0.01	0.2037
Spina bifida	0.5	0.5	0.5	0.5	0.5	0.4	0.4	0.3	-0.07	-0.1,-0.04	<.0001
Myasthenia gravis	0.4	0.4	0.4	0.4	0.3	0.4	0.4	0.3	-0.03	-0.07,0.01	0.1540
ALS/MND	0.6	0.7	0.8	0.7	0.9	0.5	0.4	0.5	-0.09	-0.16,-0.02	0.0139
Post-polio syndrome	0.3	0.0	0.3	0.3	0.3	0.3	0.3	0.3	0.13	0.01,0.24	0.0404
SMA	<0.02	0.03	<0.2	<0.2	0.02	<0.2	<0.2	0.03	0.00	-0.19,0.19	1.0000
Children											
All NMD diagnoses	4.7	5.2	4.9	4.9	4.7	3.9	4.0	5.1	-0.02	-0.06,0.01	0.1692
Cerebral palsy	0.7	1.3	0.6	0.8	0.6	0.7	0.6	0.6	-0.11	-0.2,-0.02	0.0202
Guillian Barre	0.5	0.6	0.7	0.5	0.5	0.5	0.4	0.5	-0.06	-0.1,-0.02	0.0047
Muscular dystrophy	0.4	0.5	0.6	0.4	0.4	0.3	0.3	0.6	-0.03	-0.13,0.06	0.5083
Metabolic disease	0.7	0.7	0.6	0.8	0.5	0.8	0.6	0.8	0.02	-0.04,0.08	0.6077
Myasthenia gravis	<0.2	<0.2	<0.2	0.2	<0.2	0.0	0.2	<0.2	0.11	-0.07,0.3	0.2367
ALS/MND	<0.2	<0.2	<0.2	<0.2	<0.2	<0.2	<0.2	<0.2	-0.25	-1.14,0.64	0.5812
Multiple sclerosis	0.7	0.6	0.9	0.4	0.7	0.7	0.7	1.1	0.07	-0.02,0.16	0.1394
NMD other	0.3	0.3	0.2	0.3	0.4	<0.2	0.4	0.3	0.03	-0.1,0.15	0.6996
Neuropathies	0.2	<0.2	<0.2	<0.2	0.2	<0.2	<0.2	<0.2	0.00	-0.11,0.11	1.0000
Spina bifida	1.0	0.9	1.0	1.3	1.1	0.8	0.8	0.8	-0.04	-0.1,0.01	0.1405
SMA	<0.2	<0.2	<0.2	<0.2	0.2	<0.2	0.0	<0.2	-0.07	-0.26,0.12	0.4631

^a^ Incidence/100,000 individuals whose index case was identified through ED presentation or hospital admission (i.e., excludes those identified through physician billing only).

^b^ Average change in incidence per year.

^c^ P value for linear trend.

ALS: amyotrophic lateral sclerosis; MND: motor neurone disease; NMD: neuromuscular disease (non-specific diagnosis); SMA: spinal muscular atrophy.

For children, incidence of NMD over the 7-year period (fiscal years 2008–2014) was 4.7/100,000 children considering only those identified through ED presentation or hospital admission and 56/100,000 including children identified through physician billing. Incidence remained relatively static overall (2%, 95% CI -7% to 2%, P = 0.169 linear trend) and across all age categories (Fig 3). Incidence decreased over time for multiple sclerosis, neuropathies, and myasthenia gravis (Table 2). Incidence decreased by 7% (95% CI -9% to -4%) in males but remained unchanged in females (Table C in S1 File).

### Mortality and use of palliative care

Of adults with NMD identified through hospitalization or ED presentation, 34,336 (18.5%) died over the 12-year period (16,458 (47.9%) in hospital), of whom 21,236 (61.8%) received palliative care. The proportion of individuals with NMD who died decreased from 34.8% in 2003 to 24% in 2014 (odds ratio (OR) 0.86 95% CI 0.86 to 0.87, P <.0001 for linear trend). At the same time, use of palliative care increased on average from 9.9% in 2003 to 21.5% in 2014 (OR 1.18, 95% CI 1.09 to 1.28, P <.0001 for linear trend). Of children with NMD, 1,009 (5.6%) died over the 12 year period (482 (47.8%) in hospital), of whom 507 (50.2%) received palliative care. The proportion of children that died decreased from 7.9% in 2003 to 3% in 2014 (OR 0.79 95% CI 0.76 to 0.82%, P <.0001 linear trend). At the same time, use of palliative care increased (OR 1.22, 95% CI 1.20 to 1.23, P <.0001 linear trend).

## Discussion

In this retrospective population based study of adults and children with NMD, we found prevalence of those requiring ED presentation or hospital admission to be increasing over time among both adults and children. Prevalence in adults is highest in the oldest age category but increased the most in the youngest age category, suggesting increased survival of children with NMD into adulthood. Overall incidence is decreasing in adults but remains static in children. Diseases for which the incidence remained unchanged in adults include neuropathies, muscular dystrophy, metabolic diseases, ALS, spina bifida and myasthenia gravis. Mortality was modest overall and has decreased over time. Two thirds of adults and 50% of children received palliative care. Receipt of palliative care has increased over time for adults and children.

Although there have been studies reporting point prevalence or birth prevalence of various forms of NMD [16, 17], none have been population-based studies in which trends in prevalence and incidence of NMD were ascertained. Prevalence is dependent on incidence and disease duration, the latter being related to disease resolution or death. Given the declining incidence in adults and static incidence in children and given that most forms of NMD do not resolve, the increasing prevalence observed can be attributed to increased survival. This is supported by the decline in crude mortality rates over the 12-year study period for both adults and children and the increased prevalence in both the youngest adult and child age categories.

The reduction in mortality found in our study is consistent with reports that identified an increased survival in various NMD populations including Duchenne Muscular Dystrophy [18], Guillain Barré syndrome [19], and ALS [20]. Reasons for this reduction are likely multi-factorial. For those individuals in whom the disease progresses to respiratory failure improvements to monitoring of respiratory complications, newer technologies for lung hygiene [18] and nocturnal ventilation [21] are likely contributors in addition to more timely access to specialist services, including multi-disciplinary and inter-professional specialist clinics [22].

Almost two thirds of adults and half the children that died received palliative care, with adult use increasing over time. Although the Canadian healthcare system offers advanced palliative care [23] most is delivered within hospitals rather than the community, with substantial variation in access to palliative care regardless of diagnosis related to geographic location [24]. Barriers to the utilization of palliative care for children and adolescents with NMD include poor family awareness of palliative care services and what they provide [25], service availability and cost, age-appropriateness of services, perceptions of palliative care and perceived benefit [26].

Comparison with international reports of prevalence and incidence rates is challenging due to differences in case ascertainment rates and denominator. We found the prevalence of muscular dystrophy to be 8.6/100,000 adults and 5.6/100,000 children in 2014 which is lower than other international reports and likely due to restricting our estimates to those identified through hospital admission or ED presentation and therefore likely to have more severe disease. Global estimates of the prevalence of combined muscular dystrophies derived from systematic reviews of existing studies, estimate the pooled prevalence to range from 16.1/100,000 [4] to 25.1/100,000 people [16]. We observes a slightly lower prevalence (2.1/100,000 in 2014) and incidence (0.5/100,000) of ALS compared with European ALS registers (prevalence 7–9/100,000, incidence 2.6/100,000) [27]. Our observations were more in keeping with recent US estimates of ALS prevalence (4 5.0/100,000) [5].

Study limitations include the possibility of misclassification, either under or over ascertainment of NMD due to the absence of validated algorithms for case ascertainment. We restricted reporting of disease specific prevalence and incidence to those individuals identified through hospital diagnostic codes and therefore these data likely underestimate the true population rates that include mild or early disease. However, we are able to provide population-based trends in prevalence, incidence, and mortality of adults and children with NMD requiring emergent care. Coding within the health administrative database prevented more disease specific descriptions of prevalence and incidence i.e., Duchenne muscular dystrophy, the most common muscular dystrophy. We were also unable to distinguish between acute and chronic disease. Finally, delivery of palliative care may not always be coded in the health administrative databases, as some physicians may be unaware of palliative care billing codes.

## Conclusion

In this population-based study of adults and children with NMD, we observed a rising prevalence of NMD, particularly in younger adults and children, whereas mortality is declining. The incidence of NMD is declining in adults with no change in children. These findings confirm on a population-based level the increased survival of children and adults with NMD, and will inform healthcare planning for NMD in the future. Although a reasonable proportion of adults and children received palliative care before death, we recommend further improvements in delivery and uptake of palliative care.

## Supporting information

S1 File Cohort creation codes and additional study data. (DOCX)
