# Supplementary material for: Trends in incidence, prevalence, and mortality of neuromuscular disease in Ontario, Canada: A population-based retrospective cohort study (2003-2014)
Source: PLoS One. 2019 Mar 26;14(3):e0210574. doi: 10.1371/journal.pone.0210574 (PMC6435115; doi:10.1371/journal.pone.0210574)
Supplement: S1 File — (DOCX) [file pone.0210574.s001.docx]

**Diagnostic and Physician Billing Codes by Diagnostic Group**

**ALS/MND**

ICD-9 335.2 Amyotrophic lateral sclerosis

335.21 ALS

335.22 ALS

ICD-10 G122 Motor Neuron Disease

G122.0 Amyotrophic lateral sclerosis

G12.22 Progressive bulbar palsy

G12.28 Other motor neuron disease

G12.29 Motor neuron disease, unspecified

OHIP 349 Motor neurone disease

**Cerebral palsy**

ICD-9 343 Congenital diplegia

343.1 Congenital hemiplegia

343.2 Congenital quadriplegia

343.4 Infantile hemiplegia

343.8 Other specified infantile cerebral palsy

343.9 Infantile cerebral palsy, unspecified

ICD-10 G80.0 Spastic quadriplegic cerebral palsy

G80.1 Spastic diplegic cerebral palsy

G80.2 Spastic hemiplegic cerebral palsy

G80.3 Athetoid cerebral palsy

G80.4 Ataxic cerebral palsy

G80.8 Other cerebral palsy

G80.9 Cerebral palsy, unspecified

OHIP 343 Cerebral palsy

**Muscular Dystrophy**

ICD-9 359

359 Congenital hereditary muscular dystrophy

359.1 Hereditary progressive muscular dystrophy

359.2 Myotonic disorders

359.21 Myotonic muscular dystrophy

359.22 Myotonia congenita

359.23 Myotonic chondrodystrophy

359.29 Other specified myotonic disorder

359.6 Symptomatic inflammatory myopathy in diseases classified elsewhere

359.7 Inflammatory and immune myopathies, nec

359.71 Inclusion body myositis

359.8 Other myopathies

359.81 Critical illness myopathy

359.89 Other myopathies

359.9 Myopathy, unspecified

ICD-10 G71.0 Muscular dystrophy

G71.1 Myotonic disorders

G71.11 Myotonic muscular dystrophy

G71.12 Myotonia congenita

G71.13 Myotonic chondrodystrophy

G71.19 Other specified myotonic disorders

G71.2 Congenital myopathies

G71.3 Mitochondrial myopathy, not elsewhere classified

G72.4 Inflammatory and immune myopathies, not elsewhere classified

G72.41 Inclusion body myositis (IBM)

G72.49 Other inflammatory and immune myopathies, not elsewhere classified

G72.8 Other specified myopathy keep so we dont miss any

G72.81 Critical illness myopathy

G72.89 Other specified myopathies

G72.9 Myopathy, unspecified

G73.7 Myopathy in diseases classified elsewhere

OHIP 359 Muscular dystrophies

**Myasthenia Gravis**

ICD-9 3580 Myasthenia Gravis

ICD-10 G70 Myasthenia Gravis

G70.2 Congenital and developmental myasthenia

G70.8 Other specified myoneural disorders

G70.9 Myoneural disorder, unspecified

OHIP 358 Myoneural disorders (e.g., myasthenia gravis)

**Guillain-Barre**

ICD-9 357 Guillain-Barre syndrome

ICD-10 G61.0 Guillain-Barre syndrome

**Multiple Sclerosis**

ICD-9 340 Multiple Sclerosis

ICD-10 G35 Multiple Sclerosis

G36.8 Other specified acute disseminated demyelination

G37 Other demyelinating diseases of central nervous system

G37.3 Acute transverse myelitis in demyelinating disease of central nervous system

G37.8 Other specified demyelinating diseases of central nervous system

G37.9 Demyelinating disease of central nervous system, unspecified

OHIP 340 Multiple Sclerosis

**Neuropathies**

ICD-9 337.1 Peripheral autonomic neuropathy in disorders classified elsewhere

356 Hereditary peripheral neuropathy

356.8 Other specified idiopathic peripheral neuropathy

357.8 Other inflammatory and toxic neuropathy

357.81 Chronic inflammatory demyelinating polyneuritis

357.82 Critical illness polyneuropathy

357.89 Other inflammatory and toxic neuropathy

357.9 Unspecified inflammatory and toxic neuropathy

ICD-10 G60 Hereditary and idiopathic neuropathy

G600 Hereditary motor and sensory neuropathy

G602 Neuropathy in association with hereditary ataxia

G608 Other hereditary and idiopathic neuropathies

G609 Hereditary and idiopathic neuropathy, unspecified

G61.8 Other inflammatory polyneuropathies

G61.81 Chronic inflammatory demyelinating polyneuritis

G61.89 Other inflammatory polyneuropathies

G61.9 Inflammatory polyneuropathy, unspecified

G62.8 Other specified polyneuropathies

G62.81 Critical illness polyneuropathy

G63.6 Polyneuropathy in other musculoskeletal disorders

**Postpolio Syndrome**

ICD-9 138 Late effects of acute poliomyelitis

ICD-10 G14 Postpolio syndrome

**Spina Bifida**

ICD-9 741 Spina bifida with hydrocephalus

741.00 Spina bifida with hydrocephalus, unspecified region

741.01 Spina bifida with hydrocephalus, cervical region

741.02 Spina bifida with hydrocephalus, dorsal (thoracic region)

741.03 Spina bifida with hydrocephalus, lumbar region

741.9 Spina bifida without mention of hydrocephalus

741.9 Spina bifida without mention of hydrocephalus, unspecified region

741.91 Spina bifida without mention of hydrocephalus, cervical region

741.92 Spina bifida without mention of hydrocephalus, dorsal (thoracic region

741.93 Spina bifida without mention of hydrocephalus, lumbar region

ICD-10 Q05 Spina bifida

Q05.0 Cervical spina bifida with hydrocephalus

Q05.1 Thoracic spina bifida with hydrocephalus

Q05.2 Lumbar spina bifida with hydrocephalus

Q05.3 Sacral spina bifida with hydrocephalus

Q05.4 Unspecified spina bifida with hydrocephalus

Q05.5 Cervical spina bifida without hydrocephalus

Q05.6 Thoracic spina bifida without hydrocephalus

Q05.7 Lumbar spina bifida without hydrocephalus

Q05.8 Sacral spina bifida without hydrocephalus

Q05.9 Spina bifida, unspecified

Q760 Spina bifida occulta

OHIP 741 Spina bifida, with or without hydrocephalus, meningocele, meningomyelocele

**SMA**

ICD-9 335 SMA

335.1 Spinal muscular atrophy, unspecified

335.11 SMA

335.19 SMA

ICD-10 G12 Spinal muscular atrophy and related syndromes

G120 Infantile spinal muscular atrophy, type I [Werdnig-Hoffman]

G121 Other inherited spinal muscular atrophy

G128 Other spinal muscular atrophies and related syndromes

G129 Spinal muscular atrophy, unspecified

Neuromuscular disorders-other

333.4 Huntington's chorea

G10 Huntington's disease

G11 Hereditary ataxia

G11.2 Late-onset cerebellar ataxia

G11.4 Hereditary spastic paraplegia

G11.8 Other hereditary ataxias

G11.9 Hereditary ataxia, unspecified

G13 Systemic atrophies primarily affecting central nervous system in diseases classified elsewhere

G70.8 Other specified myoneural disorders

G70.9 Myoneural disorder, unspecified

G73.3 Myasthenic syndromes in other diseases classified elsewhere

Su**pplementary Table A: Cohort creation from eligible patient population**

| **Eligible Population** | | | | | | | | | | | |
| --- | --- | --- | --- | --- | --- | --- | --- | --- | --- | --- | --- |
| **Adults** | | | | | | | | | | | |
| 2003 | 2004 | 2005 | 2006 | 2007 | 2008 | 2009 | 2010 | 2011 | 2012 | 2013 | 2014 |
| 12,396,082 | 12,555,599 | 12,711,728 | 12,866,896 | 13,026,626 | 13,186,753 | 13,368,420 | 13,506,066 | 13,631,804 | 13,750,000 | 13,862,291 | 13,960,961 |
| **Children** | | | | | | | | | | | |
| 2003 | 2004 | 2005 | 2006 | 2007 | 2008 | 2009 | 2010 | 2011 | 2012 | 2013 | 2014 |
| 3,732,504 | 3,662,140 | 3,594,046 | 3,524,908 | 3,452,352 | 3,376,831 | 3,277,497 | 3,215,338 | 3,163,691 | 3,115,593 | 3,068,247 | 3,028,410 |
| **All NMD patients identified (before exclusions)** | | | | | | | | | | | |
| **Adults** | | | | | | | | | | | |
| 2003 | 2004 | 2005 | 2006 | 2007 | 2008 | 2009 | 2010 | 2011 | 2012 | 2013 | 2014 |
| 80,502 | 131,380 | 177,965 | 219,801 | 257,797 | 294,591 | 330,938 | 366,257 | 400,070 | 431,861 | 463,995 | 497,008 |
| **Children** | | | | | | | | | | | |
| 2003 | 2004 | 2005 | 2006 | 2007 | 2008 | 2009 | 2010 | 2011 | 2012 | 2013 | 2014 |
| 13,962 | 22,244 | 29,763 | 36,904 | 43,708 | 50,301 | 56,795 | 2,787 | 68,931 | 74,730 |  |  |
| **NMD patients in cohort (after exclusions)** | | | | | | | | | | | |
| **Adults** | | | | | | | | | | | |
| 2003 | 2004 | 2005 | 2006 | 2007 | 2008 | 2009 | 2010 | 2011 | 2012 | 2013 | 2014 |
| 53,184 | 83,475 | 110,833 | 135,777 | 158,447 | 180,182 | 201,771 | 222,715 | 242,223 | 260,179 | 277,288 | 299,259 |
| **Children** | | | | | | | | | | | |
| 2003 | 2004 | 2005 | 2006 | 2007 | 2008 | 2009 | 2010 | 2011 | 2012 | 2013 | 2014 |
| 7,153 | 9,854 | 12,225 | 14,357 | 16,323 | 18,119 | 19,839 | 21,462 | 23,206 | 24,840 | 26,300 | 27,823 |
| NOTE: all counts are prevalent cases | | |  |  |  |  |  |  |  |  |  |

a Exclusions:

1. death before April 1 in the year

2. not eligible to the OHIP

3. not Ontario residents

**Table B Prevalence by age and sex**

| Overall (yrs) | | 2003 | 2004 | 2005 | 2006 | 2007 | 2008 | 2009 | 2010 | 2011 | 2012 | 2013 | 2014 | Rate^a^ | 95% CI | P^b^ |
| --- | --- | --- | --- | --- | --- | --- | --- | --- | --- | --- | --- | --- | --- | --- | --- | --- |
| Adults | | | | | | | | | | | | | |  |  |  |
| Age group |  |  |  |  |  |  |  |  |  |  |  |  |  |  |  |  |
| 65+ | 126.7 | 71.2 | 98.4 | 114.0 | 126.5 | 131.2 | 137.2 | 140.3 | 144.1 | 143.8 | 142.1 | 136.4 | 135.5 | 0.04 | 0.02,0.05 | <.0001 |
| 40 - 64 | 72.9 | 30.7 | 44.0 | 53.4 | 61.6 | 68.6 | 74.5 | 79.2 | 84.7 | 88.7 | 93.0 | 96.3 | 100.3 | 0.08 | 0.07,0.09 | <.0001 |
| 18 - 39 | 50.8 | 16.8 | 25.6 | 33.1 | 39.8 | 45.3 | 50.0 | 53.8 | 59.2 | 64.4 | 69.4 | 74.3 | 77.7 | 0.11 | 0.10,0.11 | <.0001 |
| Sex |  |  |  |  |  |  |  |  |  |  |  |  |  |  |  |  |
| Female | 78.9 | 32.7 | 47.8 | 58.0 | 67.2 | 74.3 | 80.7 | 85.6 | 91.5 | 96.0 | 100.6 | 104.0 | 108.0 | 0.08 | 0.07,0.09 | <.0001 |
| Male | 66.4 | 27.9 | 39.9 | 49.3 | 57.2 | 62.9 | 68.0 | 72.0 | 77.3 | 81.3 | 84.7 | 86.8 | 89.3 | 0.08 | 0.07,0.09 | <.0001 |
| Children | | | | | | | | | | | | | |  |  |  |
| Age group |  |  |  |  |  |  |  |  |  |  |  |  |  |  |  |  |
| 0 - 5 | 65.5 | 24.7 | 34.1 | 41.8 | 45.9 | 53.5 | 60.8 | 68.4 | 74.7 | 83.0 | 89.9 | 97.2 | 111.5 | 0.11 | 0.11,0.12 | <.0001 |
| 6 - 10 | 39.8 | 20.5 | 28.9 | 32.2 | 35.6 | 38.3 | 40.8 | 43.3 | 45.4 | 47.1 | 47.6 | 48.4 | 48.9 | 0.06 | 0.05,0.07 | <.0001 |
| 11 - 17 | 45.4 | 18.6 | 25.9 | 30.7 | 34.9 | 38.7 | 43.2 | 48.4 | 53.3 | 57.4 | 60.5 | 64.5 | 68.1 | 0.10 | 0.09,0.10 | <.0001 |
| Sex |  |  |  |  |  |  |  |  |  |  |  |  |  |  |  |  |
| Male | 54.3 | 23.1 | 31.7 | 37.7 | 41.5 | 46.6 | 52.1 | 58.1 | 63.0 | 67.9 | 72.3 | 75.9 | 81.4 | 0.09 | 0.09,0.10 | <.0001 |
| Female | 45.4 | 18.5 | 26.2 | 30.7 | 35.0 | 39.2 | 43.5 | 48.2 | 52.4 | 57.2 | 59.9 | 64.4 | 70.1 | 0.10 | 0.09,0.10 | <.0001 |

a Average change in prevalence per year.

b P value for linear trend.

**Table C Incidence by age and sex**

| Overall (yrs) | | 2008 | 2009 | 2010 | 2011 | 2012 | 2013 | 2014 | Rate^a^ | 95% CI | P^b^ |
| --- | --- | --- | --- | --- | --- | --- | --- | --- | --- | --- | --- |
| Adults | | | | | | | | | | | |
| Age group |  |  |  |  |  |  |  |  |  |  |  |
| 65+ | 26.1 | 30.4 | 28.5 | 28.3 | 27.2 | 23.4 | 22.0 | 22.7 | -0.06 | -0.07,-0.04 | <.0001 |
| 40 - 64 | 9.5 | 11.0 | 9.9 | 10.4 | 9.2 | 8.9 | 8.5 | 8.4 | -0.05 | -0.06,-0.03 | <.0001 |
| 18 – 39 | 5.2 | 5.4 | 4.6 | 5.6 | 5.5 | 5.3 | 5.1 | 4.7 | -0.01 | -0.03,0.02 | 0.4462 |
| Sex |  |  |  |  |  |  |  |  |  |  |  |
| Female | 10.9 | 12.3 | 11.0 | 11.6 | 10.7 | 10.6 | 10.0 | 10.3 | -0.03 | -0.04,-0.02 | <.0001 |
| Male | 10.3 | 11.3 | 10.5 | 11.3 | 10.9 | 9.5 | 9.4 | 9.2 | -0.04 | -0.05,-0.02 | <.0001 |
| Children | | | | | | | | | | | |
| Age group |  |  |  |  |  |  |  |  |  |  |  |
| 0 – 5 | 8.8 | 9.7 | 9.1 | 7.9 | 8.7 | 8.7 | 7.1 | 10.2 | -0.01 | -0.05,0.03 | 0.7383 |
| 6 - 10 | 2.1 | 2.6 | 2.8 | 2.7 | 1.9 | 1.2 | 1.4 | 2.4 | -0.08 | -0.17,0.00 | 0.0572 |
| 11-17 | 3.3 | 3.8 | 3.2 | 4.0 | 3.5 | 2.1 | 3.3 | 3.3 | -0.03 | -0.09,0.02 | 0.2411 |
| Sex |  |  |  |  |  |  |  |  |  |  |  |
| Male | 4.9 | 6.0 | 5.6 | 5.2 | 4.5 | 4.6 | 3.6 | 4.5 | -0.07 | -0.09,-0.04 | <.0001 |
| Female | 4.5 | 4.5 | 4.2 | 4.5 | 4.9 | 3.3 | 4.4 | 5.8 | 0.02 | -0.03,0.08 | 0.3744 |

a Average change in incidence per year.

b P value for linear trend.
